# A Real-Time FPGA-Based Metaheuristic Processor to Efficiently Simulate a New Variant of the PSO Algorithm

**DOI:** 10.3390/mi14040809

**Published:** 2023-03-31

**Authors:** Esteban Anides, Guillermo Salinas, Eduardo Pichardo, Juan G. Avalos, Giovanny Sánchez, Juan C. Sánchez, Gabriel Sánchez, Eduardo Vazquez, Linda K. Toscano

**Affiliations:** Instituto Politécnico Nacional, ESIME Culhuacan, Av. Santa Ana No. 1000, Ciudad de México 04260, Mexico

**Keywords:** FPGA, parallel metaheuristic processor, particle swarm optimization, Markovian switching technique, AEC system, spiking neural P systems

## Abstract

Nowadays, high-performance audio communication devices demand superior audio quality. To improve the audio quality, several authors have developed acoustic echo cancellers based on particle swarm optimization algorithms (PSO). However, its performance is reduced significantly since the PSO algorithm suffers from premature convergence. To overcome this issue, we propose a new variant of the PSO algorithm based on the Markovian switching technique. Furthermore, the proposed algorithm has a mechanism to dynamically adjust the population size over the filtering process. In this way, the proposed algorithm exhibits great performance by reducing its computational cost significantly. To adequately implement the proposed algorithm in a Stratix IV GX EP4SGX530 FPGA, we present for the first time, the development of a parallel metaheuristic processor, in which each processing core simulates the different number of particles by using the time-multiplexing technique. In this way, the variation of the size of the population can be effective. Therefore, the properties of the proposed algorithm along with the proposed parallel hardware architecture potentially allow the development of high-performance acoustic echo canceller (AEC) systems.

## 1. Introduction

Over the last ten years, tremendous efforts have been made to develop high-performance acoustic echo cancellers, which can offer high-quality and realistic sound needed in the newest acoustic communications. In particular, some authors have used the PSO algorithm as an adaptive echo canceling algorithm since this can be easily implemented and exhibits a fast convergence rate [1]. For example, Mahbub et al. [1,2] presented an AEC system based on the PSO to compute the error minimization in the frequency domain and time domain, respectively. Pichardo et al. [3] introduced a convex combination to improve the performance of the digital filter at the cost of increasing the overall computational cost. Recently, Kimoto et al. [4] introduced a multichannel adaptive echo-canceling algorithm based on PSO. Specifically, their technique considers a pre-processing of the input signals. Despite achieving these advanced approaches, there are still remaining tasks to significantly improve the performance of these systems in terms of echo return loss enhancement (ERLE) and convergence rate. Unfortunately, PSO suffers from premature convergence problems, especially in the case of multi-modal optimization problems. This reduces its performance since it losses the ability to find the optimal solution. To deal with this, several authors have proposed modifications to the conventional PSO [5,6,7,8,9,10]. However, a small number of solutions have been applied to adaptive acoustic echo cancellers. On the other hand, the implementation of the PSO algorithm in FPGA devices, to simulate AEC systems, faces great challenges to build optimal hardware architectures. To date, several hardware architectures have been developed to implement the PSO algorithm to be applied in adaptive filtering [11,12,13]. However, none of these architectures have been proposed to simulate AEC systems. Here, we present for the first time, the implementation of the PSO algorithm in an FPGA for AEC systems. Specifically, we include the Markovian switching technique [14] into the conventional PSO algorithm to create a high-performance AEC system since considers quick convergence to the global optimum and also keeps the swarm global search simultaneously by taking advantage of the current search information.

From the engineering point of view, the implementation of the AEC systems based on the PSO algorithm requires a large number of particles. As a consequence, a large area of consumption is required. To overcome this, we proposed criteria to dynamically decrease the number of particles over the filtering process. In addition, we use the block-processing scheme to easily implement the proposed algorithm in a parallel hardware architecture.

## 2. Proposed Markov Switching PSO Algorithm

**Figure 1:**
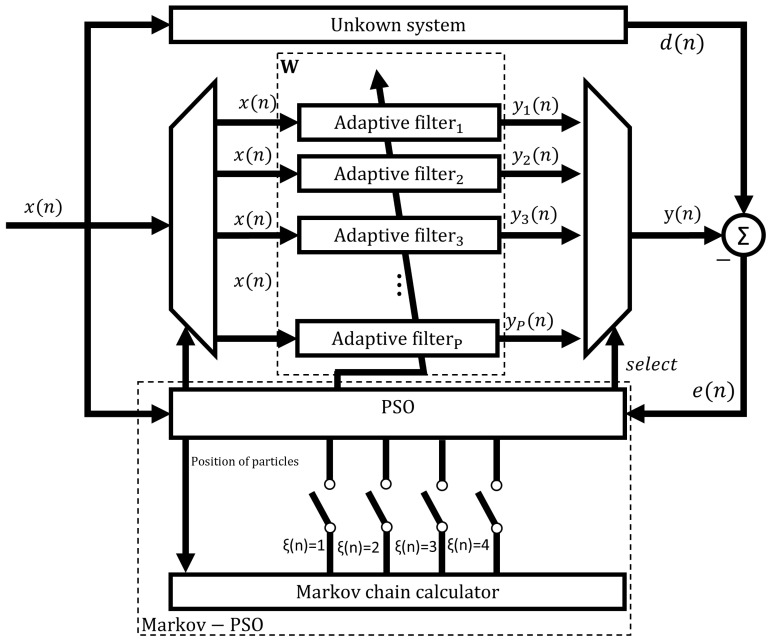
Structure of the proposed Markov switching PSO algorithm applied to adaptive filtering.

**Figure 2:**
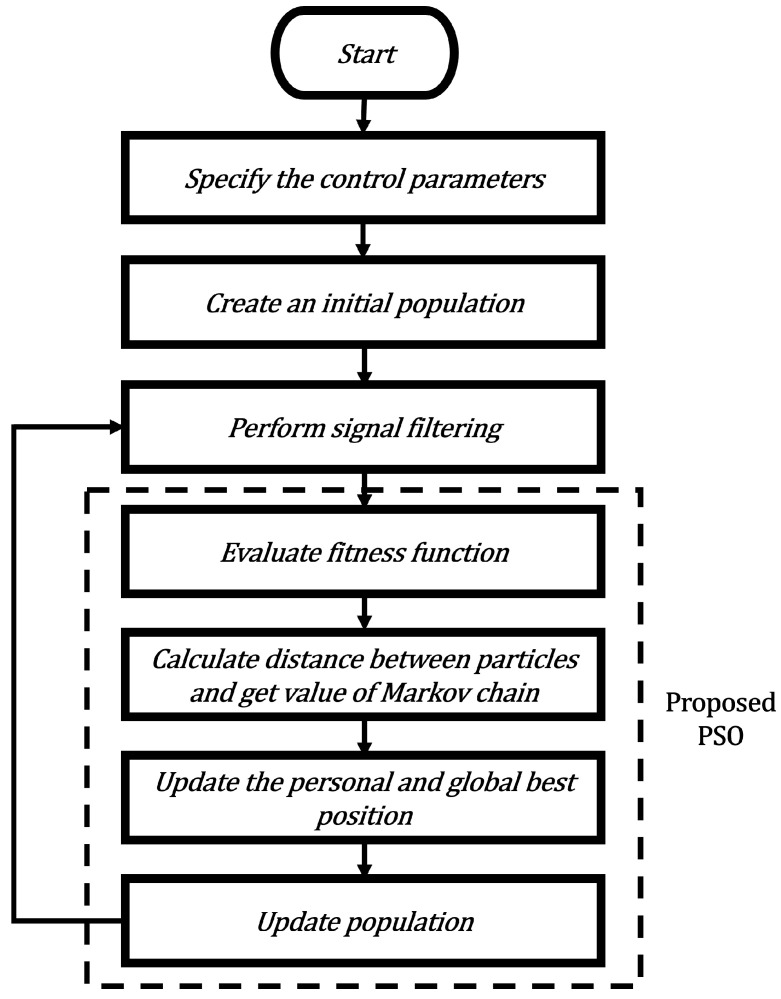
Flowchart of the proposed Markov switching PSO algorithm.

In this section, we present a new variant of the PSO to improve search performance. Specifically, we dynamically adjust the velocity of the particle according to an evolutionary factor. In this manner, the premature convergence of the PSO can be prevented and this can be also especially useful in dealing with multi-modal and high-dimensional problems. Figure 1 shows the structure of the proposed variant of the PSO algorithm applied to adaptive filtering and Figure 2 shows the required steps to perform the proposed PSO algorithm.

To perform the proposed PSO algorithm, the following steps are required:

- *Specification of the control parameters*. Here, the proposed Markov switching PSO algorithm has a population matrix W with *P* adaptive filters, where each particle denotes an adaptive filter, as shown in Equation (1). Here, the order *N* of each adaptive filter determines the dimension of each particle. Therefore, the whole population is defined as follows:
  (1) $$W=\left[\begin{array}{cccc} w_{1}^{1} & w_{1}^{2} & \cdots & w_{1}^{P}\\ w_{2}^{1} & w_{2}^{2} & \cdots & w_{2}^{P}\\ \vdots & \vdots & \ddots & \vdots \\ w_{N}^{1} & w_{N}^{2} & \cdots & w_{N}^{P} \end{array}\right]$$
- *Creation of the initial population*. At the first iteration n=1, the position $w_{i}\left(n\right)$ of each particle is initialized, where i=1,2,⋯,P.
  (2) $$w_{i}\left(n\right)=\left(ub-lb\right)\cdot r+lb$$
  where r denotes a Gaussian process of length, *N*. The value lb is a lower bound and ub is an upper bound.
- *Calculation of the signal filtering*. The calculation of signal counteraction or also called residual noise e(n) is given by
  (3) $$e_{i}\left(n\right)=d\left(n\right)+y_{i}\left(n\right)$$
  d(n) denotes the desired signal and y(n) the filter output of the *i*-th filter.
- *Evaluation of the fitness function*. To compute the best position, the PSO algorithm uses the mean squared error (MSE) of each *P* error signal as a fitness function of each adaptive filter. The evaluation of the position $w_{i}\left(n\right)$ can be computed as follows:
  (4) $$f_{i}\left(n\right)=\frac{1}{N}\sum_{k=1}^{N}e_{i}^{2}\left(k\right)$$
- *Calculation of the distance between particles and the obtention of the value of Markov chain*. Here, the velocity and position are obtained by using the following equations:
  (5) $$v_{i}\left(n\right)=\phi \cdot v_{i}\left(n-1\right)+c_{1}\left(\xi \left(n\right)\right)\cdot r_{1}\left[w_{pbest_{i}}-w_{i}\left(n\right)\right]+c_{2}\left(\xi \left(n\right)\right)\cdot r_{2}\left[w_{gbest}-w_{i}\left(n\right)\right]$$
  (6) $$w_{i}\left(n\right)=w_{i}\left(n-1\right)+v_{i}\left(n\right)$$
  where $r_{1}$ and $r_{2}$ are the vectors of random numbers of length *N*, $w_{pbest_{i}}$ and $w_{gbest}$ are the personal best position and global best position, respectively, and ϕ the inertia weight. Here, we define $r_{1}$ and $r_{2}$ in the interval [0, 1]. $c_{1}\left(\xi \left(n\right)\right)$ and $c_{2}\left(\xi \left(n\right)\right)$ are the acceleration coefficients determined by a non-homogeneous Markov chain ξ(n)(n≥0). The value of the Markov chain is taken in a finite state space: S={1,2,⋯,L}. $\Pi \left(n\right)={\left(\pi_{ij}\left(n\right)\right)}_{LxL}$, where Π(n) denotes the probability transition matrix of the Markov chain, where $\pi_{ij}\left(n\right)\geq 0\left(i,j\in S\right)$ and $\sum_{j=1}^{N}\pi_{ij}\left(n\right)=1$. It is important to keep in mind that the matrix Π is dynamically adjusted by evaluating an evolutionary factor ($E_{f}$) [14] according to the population distribution properties [15]. Based on these characteristics, the $E_{f}$ approach can be exploited at the maximum to define four states: convergence, exploration, exploitation and jumping out. In particular, these four states are, respectively, represented by ξ(n)=1, ξ(n)=2, ξ(n)=3 and ξ(n)=4 in the Markov chain.
  The average distance, $d_{i}$, between each particle and the other particles is computed as follows:
  (7) $$d_{i}\left(n\right)=\frac{1}{P}\sum_{j=1}^{P}\sqrt{\sum_{k=1}^{N}{\left(x_{i}\left(k\right)-x_{j}\left(k\right)\right)}^{2}}$$
  where *P* and *N* denotes the swarm size and the dimensions of each particle, respectively. Hence, the evolutionary factor $E_{f}$ can be obtained as follows [15]:
  (8) $$E_{f}=\frac{d_{g}-d_{min}}{d_{max}-d_{min}}$$
  where $d_{g}$ represents the globally best particle among $d_{i}$. $d_{max}$ and $d_{min}$ are the maximum and minimum distances in $d_{i}$, respectively.
  Here, we obtain the value of the Markov chain, which is based on the value of evolutionary factor $E_{f}$, as follows [14]:
  (9) $$\xi \left(n\right)=\left\{\begin{array}{cc} 1, & 0\leq E_{f}<0.25,\\ 2, & 0\leq E_{f}<0.5,\\ 3, & 0\leq E_{f}<0.75,\\ 4, & 0\leq E_{f}<1, \end{array}\right.$$
  where the probability transition matrix is given by:
  (10) $$\Pi =\left(\begin{array}{cccc} \chi & 1-\chi & 0 & 0\\ \frac{1-\chi }{2} & \chi & \frac{1-\chi }{2} & 0\\ 0 & \frac{1-\chi }{2} & \chi & \frac{1-\chi }{2}\\ 0 & 0 & 1-\chi & \chi \end{array}\right)$$
  Based on the probability distribution matrix Π, the Markov process may switch its state at the next iteration. To guarantee the classification accuracy and the search diversity, the value of the probability χ is equal to 0.9 [14]. Here, the initial values of acceleration coefficients $c_{1}$ and $c_{2}$ are selected by trial-and-error for all states in order to guarantee the best performance of the purposed algorithm. Table 1 shows their values based on the evolutionary state, which are automatically adjusted.
- *Update the personal and global best position*. To get the value of the personal best $w_{pbest_{i}}\left(n\right)$, a comparison between the current value of $f_{i}\left[w_{i}\left(n\right)\right]$ and the value of $f[w_{pbest_{i}}\left(n-1\right)]$ is performed as follows:
  (11) $$w_{pbest_{i}}\left(n\right)=\left\{\begin{array}{cc} w_{i}\left(n\right), & \mathrm{if}f_{i}\left[w_{i}\left(n\right)\right]<f\left[w_{pbest_{i}}\left(n-1\right)\right]\\ w_{pbest_{i}}\left(n-1\right), & \mathrm{otherwise} \end{array}\right.$$
  The $w_{i}\left(1\right)$ defined as $w_{pbest_{i}}\left(1\right)$ is used to calculate Equation (11) at the first generation. To calculate the global best position, $w_{gbest}$, we compare the result of $f[w_{pbest_{min}}\left(n\right)]$ with the evaluation of the best global position $f[w_{gbest}\left(n-1\right)]$, where $w_{pbest_{min}}\left(n\right)=w_{pbest_{g}}\left(n\right)$, $g=argmin_{1\leq i\leq P}\left\{w_{pbest_{j,i}}\left(n\right)\right\}$. The computation of the global best position, $w_{gbest}$ is obtained as follows:
  (12) $$w_{gbest}\left(n\right)=\left\{\begin{array}{cc} w_{pbest_{min}}\left(n\right), & \mathrm{if}f\left[w_{pbest_{min}}\left(n\right)\right]<f\left[w_{gbest}\left(n-1\right)\right]\\ w_{gbest}\left(n-1\right), & \mathrm{otherwise} \end{array}\right.$$
- *Update population*. Equations (5) and (6) are used to update the velocity and position of each particle, respectively, and Equation (13), which is in the function of the power of the instantaneous error, is used to update the population size.
  (13) $$P=\left\lfloor \frac{2(P_{max}-P_{min})}{1+e^{-e{\left(n\right)}^{2}}}-\left(P_{max}-P_{min}\right)\right\rfloor +P_{min}$$
  where $P_{max}$ and $P_{min}$ are the maximum and minimum number of particles, respectively.

## 3. Pure Software Implementation

**Figure 3:**
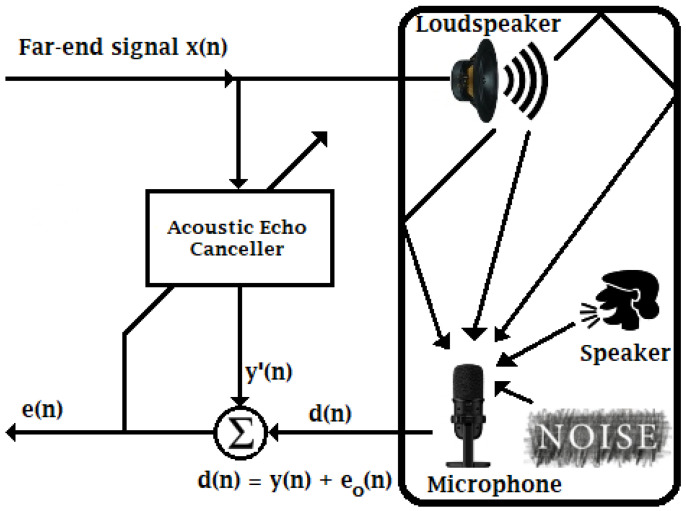
Structure of Acoustic Echo Canceller.

Before implementing the proposed Markov switching PSO algorithm in parallel hardware architectures, we simulate it in Matlab software for testing and comparison purposes. Here, we use the AEC structure, in which the existing approaches and the proposed adaptive filter are used, as shown in Figure 3. As can be observed, x(n) is the far-end input signal, e(n) denotes the residual echo signal, d(n) represents the sum of the echo signal, y(n), and the background noise, $e_{0}\left(n\right)$.

**Figure 4:**
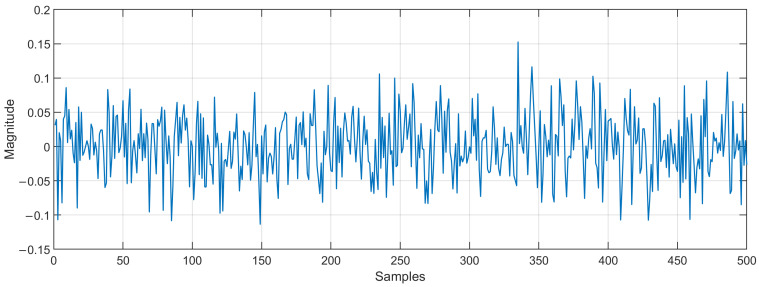
Impulse response of the acoustic echo path.

To simulate the proposed Markov switching PSO algorithm, we consider the following conditions:

- We use an impulse response as an echo path obtained from the ITU-T G168 recommendation [16]. This echo path is modeled using *N* = 500 coefficients, as shown in Figure 4.
- The echo signal is mixed with white Gaussian noise (SNR = 20 dB).
- The input signal is an AR(1) process, which is produced by filtering white Gaussian noise by means of the system $\frac{1}{\left(1-0.95z^{-1}\right)}$.
- In the proposed Markov switching PSO algorithm, the swarm size is defined in the range of 100–20 particles, while the swarm size, which is used in the simulation of an existing approach, is set to 100.
- To probe the tracking capabilities of the proposed Markov switching PSO algorithm, 4 different experiments were simulated: (1) Changing SNR from 20 dB to 10 dB in the middle of iterations, (2) causing an abrupt change to the impulse response of the acoustic echo path in the middle of the adaptive filtering process by multiplying the acoustic path by −1, (3) causing an abrupt change to the impulse response of the acoustic echo path in the middle of the adaptive filtering process by shifting the acoustic path, and (4) simulating a double talk-scenario at the middle of iterations.
- Acceleration coefficients of the conventional PSO were selected to obtain the best performance.
- The maximum number of iterations is set to 4,000,000.
- We verify the performance of the proposed algorithm in terms of echo return loss enhancement, ($ERLE=10log_{10}\left(\frac{d{\left(n\right)}^{2}}{e{\left(n\right)}^{2}}\right)$).

**Figure 5:**
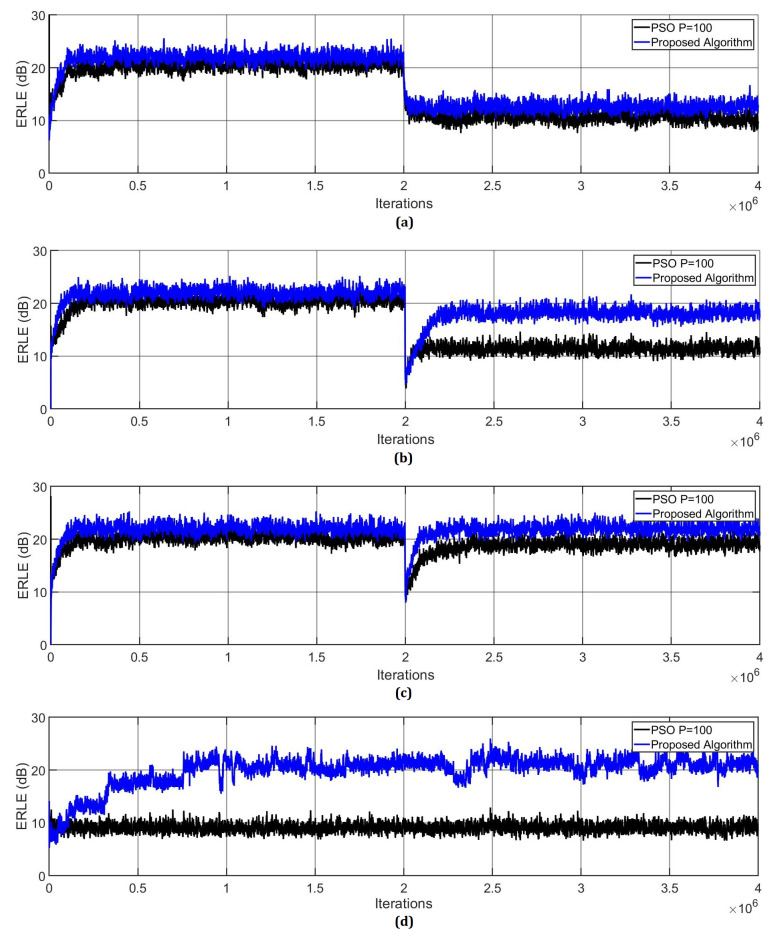
ERLE of the proposed Markov switching PSO algorithm and conventional PSO algorithms by computing the AR(1) process input signal. (**a**) Changing the SNR from 20 dB to 10 dB at the middle of the iterations. (**b**) Multiplying the acoustic path by −1 in the middle of the adaptive filtering process. (**c**) Shifting the acoustic path in the middle of the adaptive filtering process. (**d**) Simulating the double-talk scenario.

Here, we simulate the conventional PSO [17] and the proposed Markov switching PSO algorithm to compare their performance. As can be observed from Figure 5, the proposed algorithm shows better performance in comparison with the conventional PSO in terms of echo return loss enhancement (ERLE) and convergence speed.

To make a coherent comparison between the proposed algorithm and existing algorithms we carried out an experiment in which the acoustic path is multiplied by −1 midway through the iterations. The algorithms that were simulated for this comparison and their tuning parameters are described in the following list:

1. Grey wolf optimization (GWO) [18]
  - Population size =50
  - lower bound =−1
  - Upper bound =1
  - *a* decreases linearly from 2 to 0
2. PSO [19]
  - Population size =100
  - Lower bound =−1
  - Upper bound =1
  - Acceleration coefficient, $c_{1}=1.6$
  - Acceleration coefficient, $c_{2}=1$
  - Inertia weight =0.8
3. Differential evolution (DE) [20]
  - Population size =50
  - Lower bound =−1
  - Upper bound =1
  - Crossover rate =0.35
  - Scaling factor =0.8
  - Combination factor =0.25
4. Artificial bee colony optimization (ABC) [21]
  - Population size =50
  - Lower bound =−1
  - Upper bound =1
  - Evaporation parameter =0.1
  - Pheromone =0.6
5. Hybrid PSO–LMS [22]
  - Population size =60
  - Lower bound =−1
  - Upper bound =1
  - Acceleration coefficient, $c_{1}=0.00005$
  - Acceleration coefficient, $c_{2}=1.2$
  - Inertia weight =1
  - Convergence factor $=1\times 10^{-9}$
6. Modified ABC (MABC) [23]
  - Population size =50
  - Lower bound =−1
  - Upper bound =1
  - Evaporation parameter =0.1
  - Pheromone =0.6
  - Convergence factor $=3\times 10^{-5}$

**Figure 6:**
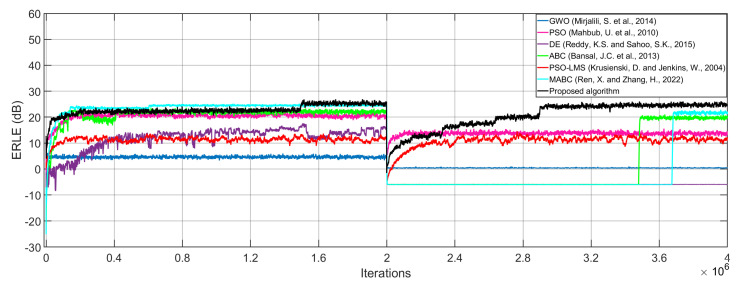
ERLE of the proposed Markov switching PSO algorithm and existing approaches [18,19,20,21,22,23] by computing the AR(1) process input signal when multiplying the acoustic path by −1 at the middle of the adaptive filtering process.

As can be observed from Figure 6, the proposed algorithm shows the best performance in terms of convergence speed and ERLE level. In addition, the proposed algorithm requires a lower computational cost when compared with the GWO, PSO, ABC, PSO-LMS and MABC algorithms, as shown in Table 2. To obtain these data, we consider a double-talk scenario, in which the proposed Markov switching PSO algorithm requires fewer multiplications and additions when compared with most of the existing algorithms during the whole simulation. The reason for this is that the number of particles, *P*, which are used to model the proposed Markov switching PSO algorithm, is reduced during the filtering process. Specifically, we initially use 100 particles, after some iterations, this number is reduced to 20 particles. In contrast, the existing approaches need a larger population to obtain acceptable performance at the cost of increasing the computational burden. In AEC applications, this aspect is crucial because one of the objectives of the proposed Markov switching PSO algorithm is to improve the computational cost without losing performance and this is possible by using the Markov Switching technique.

## 4. Hardware Implementation

**Figure 7:**
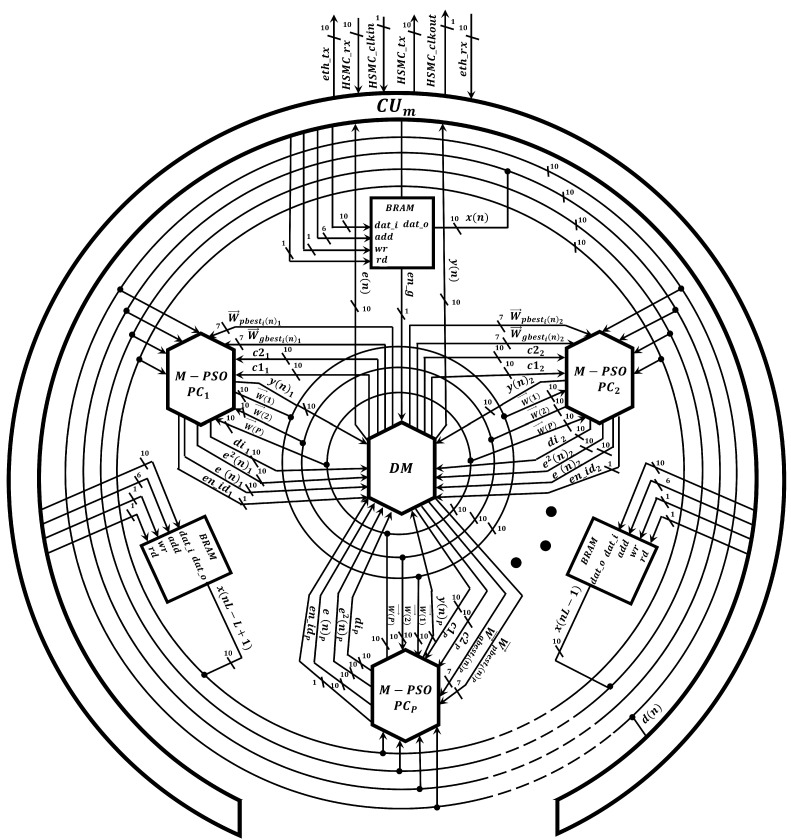
Digital implementation of the proposed Markov switching PSO algorithm in the parallel metaheuristic processor.

**Figure 8:**
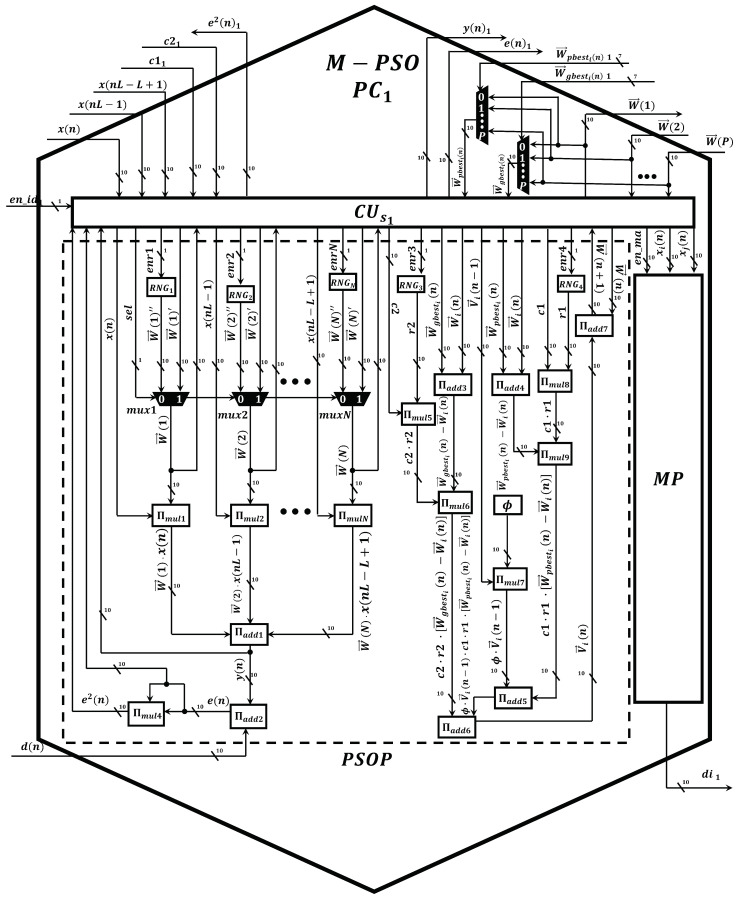
Digital circuit scheme of the Markov-PSO processing core.

**Figure 9:**
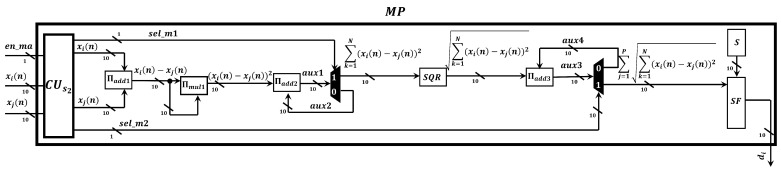
Digital circuit scheme of the Markov proccesor.

Once the performance of the proposed Markov-PSO adaptive filter was verified, we develop a parallel metaheuristic processor to simulate it at high processing speeds by expanding the minimum area consumption. To achieve a low area consumption; first, we use the time multiplexing technique to simulate particles virtually; second, we use optimized neural multipliers and adders since is the most demanding arithmetic circuit in terms of area and processing speed. In addition, the use of these circuits has allowed us to optimize the processing time since each operation is performed at a single clock cycle [24]. As can be observed from Figure 7, the proposed parallel metaheuristic processor is mainly composed of the following components:

- *Markov-PSO processing core, M-PSO PC*. This represents the basic processing element to compute the signal-filtering process and update the population. The proposed M-PSO PC mainly uses neural multipliers $\Pi_{mul}$ [24] and adders $\Pi_{add}$ [24]. Additionally, this circuit has a slave control unit $CU_{s1}$, pseudo-random number generators, RNG, and a Markov processor core, MP (Figure 8). In particular, the MP core is in charge of performing the calculation of the distance between particles by means of the optimized square root circuit [25], as shown in Figure 9.
- *A master control unit, $CU_{m}$*. This module is in charge of controlling the data flow and synchronization. Specifically, this component performs the time multiplexing technique to simulate several particles at different times by using the same *M-PSO PC*. In addition, this component sends the control signals to store the input samples, x(n), in the BRAMs. In this way, the block-processing technique can be properly implemented.
- *Distribution module, DM*. The main function of this component is to evaluate and indicate the personal and global best. Therefore, this component transfers this information to each *M-PSO PC* in parallel. Therefore, the update process can be done at high processing speeds.

It should be noted that we presented a neural adder circuit, $\prod_{add}$, and a neural multiplier, $\prod_{mul}$, to perform addition and multiplication of fixed-point numbers, respectively, in [24]. To implement the AEC system in an FPGA device, the fixed point representation is highly demanded since the simulation of metaheuristic algorithms requires high-precision calculations. Therefore, we created advanced neurons, which are based on spiking neural P systems, by improving their structural and functional capabilities. In particular, we used cutting-edge variants of the SN P systems, such as anti-spikes, dendritic trunk, dendritic delays and rules on the synapses. As a result, we create high-precision neural adders and multipliers by employing a low number of synapses and neurons with simple spiking rules. In general, both circuits exhibit the following features:

- *Scalability*. These circuits can process numbers with any required length by only adding neurons in a regular and homogenous neural structure.
- *Compactness*. To obtain a great improvement in terms of area, we designed the circuit by using a low number of neurons and synapses. Specially, we optimized the number of synapses since the routing of a large number of synaptic connections creates place and routing problems, especially when they are implemented in advanced FPGAs.
- *High performance*. In this application, the real-time filtering process is highly demanded. Therefore, we achieved neural multiplier and adder to perform their respective operations by expending a single and ten clock cycles, respectively.

**Figure 10:**
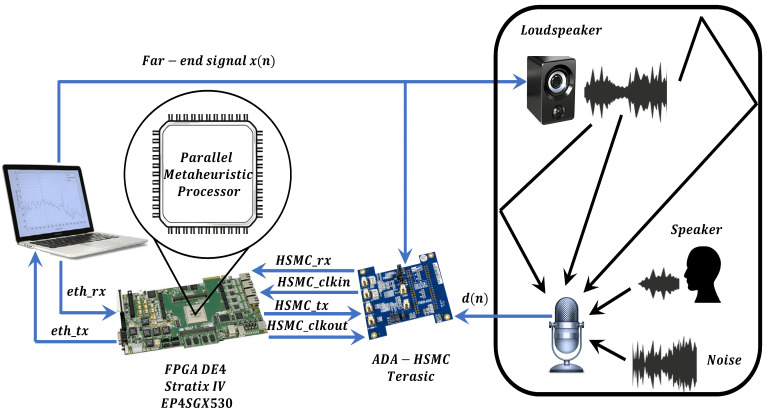
General scheme of the components of the AEC system.

Once the proposed parallel metaheuristic processor was debugged, we integrate it into the structure of the AEC system to validate its performance, as shown in Figure 10.

**Figure 11:**
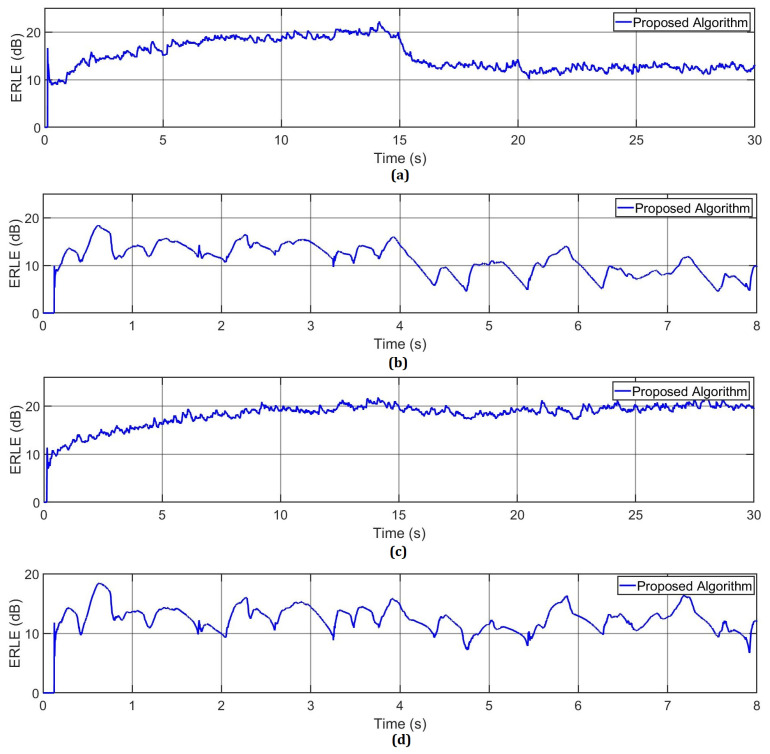
(**a**) AR(1) process and (**b**) speech signal used in a single-talk scenario; (**c**) AR(1) process and (**d**) speech signal used in a double-talk scenario.

To demonstrate the computational capabilities of the meta-heuristic processor, we develop two sets of experiments. Particularly, we simulate single-talk and double-talk scenarios by employing two different input signals, x(n). In addition, under these scenarios, the proposed metaheuristic processor computes the AR(1) process and the speech signal by considering an under-modeling case. Specifically, we employ 512 coefficients to model the adaptive filter, while the echo path [26] is configured by using 1024 coefficients. In real-world echo noise applications, the performance of the AEC can be crucially decreased by the variations of background noise. For a single-talk scenario, we decreased the SNR from 20 to 10 dB in the middle of the iterations, as shown in Figure 11. It should be noted that the background noise variation does not affect the performance of the proposed algorithm. On the other hand, the proposed metaheuristic processor was implemented in Stratix IV GX EP4SGX530 FPGA. Here, this implementation, which involves the use of eight BRAMs and 20 *M-PSO PCs*, requires 384,748 LEs. This represents 72.429% of the total area of the FPGA. In this way, we can simulate 100 particles virtually since each *M-PSO PC* simulates five particles serially. The processing time to simulate all of these particles is 89.1 μs, which are obtained by multiplying the number of clock cycles (11,143), which are obtained by means of Equation (14), by the system clock period (8 ns). It should be noted that the required processing time in the FPGA device is less in comparison when this algorithm is simulated in a server, which includes a Xeon E5-2630 processor working at 2.6 GHz and 64 Gb RAM, since the simulation of the algorithm on this computer requires 1.47 ms, considering the simulation of 100 particles. This can be considered the worse case since the number of particles decreases over the filtering process. As a consequence, the processing time also decreases. This factor is vital in the simulation of real-time AEC systems since the maximum latency of the system is 125 μs, i.e., the input signal is sampled at 8 KHz. On the other hand, the simulation of the proposed Markov-PSO adaptive filter consumes up to 328 mW, by considering the worst case (one hundred particles). After observing the results of the above experiments, we prove that the metaheuristic processor is capable of processing a variable number of particles to perform the proposed Markov switching PSO algorithm at high processing speeds.

(14) $$N_{cc}=128+\left(y-12+1+x\right)\cdot 5+\left(y-12+1+x\right)\cdot \frac{x}{20}+\left(1000+1\right)\cdot \frac{x}{20}$$

where *y* represents the number of coefficients and *x* depicts the number of particles.

## 5. Conclusions

In this work, we present, for the first time the development of a high-speed and compact FPGA-based parallel metaheuristic process to efficiently simulate a new variant of the PSO algorithm based on the Markovian switching technique. Here, we grouped our contributions as follows:

- *From the AEC model point of view*. In this work, we made intensive efforts to reduce the computational cost of the AEC systems to be implemented in resource-constrained devices. In addition, we significantly improve the convergence properties of these systems by using an improved metaheuristic swarm intelligence method to be used in practical acoustic environments. Specifically, we present a new variant of the PSO algorithm based on the Markovian switching technique. The use of this technique has allowed us to guarantee a higher convergence rate and higher ERLE in comparison when the conventional PSO algorithm is used. To make feasible the implementation of the proposed variant of the PSO algorithm in embedded devices, we use the block-processing scheme. In this way, the proposed algorithm can be easily implemented in parallel hardware architectures. As a consequence, it can be simulated at high processing speeds. In addition, we significantly reduce the computational cost of the proposed conventional PSO algorithm. To achieve this aim, we propose a method to dynamically decrease the number of particles of this new variant of the PSO algorithm over the filtering process.
- *From the digital point of view*. In this work, we present for the first time, the development of a parallel hardware architecture to simulate a variable number of particles by using the proposed time-multiplexing control scheme. In this way, we properly implement the proposed Markov switching PSO algorithm, in which the number of particles decreases according to the simulation needs, in a Stratix IV GX EP4SGX530 FPGA.

Finally, we carry out several experiments to prove that the proposed Markov switching PSO algorithm along with new techniques potentially allows the creation of practical and real-time AEC processing tools.

## Figures and Tables

**Table 1:** Strategies for selecting $c_{1}$ and $c_{2}$.

State	Mode	$c_{1}$	$c_{2}$
Convergence	ξ(n)=1	2	2
Exploitation	ξ(n)=2	2.1	1.9
Exploration	ξ(n)=3	2.2	1.8
Jumping-out	ξ(n)=4	1.8	2.2

**Table 2:** Number of additions and multiplications required by the conventional PSO algorithm [17] and the proposed algorithm.

Operation	Algorithm	Equation	Number of Operations
Addition	GWO	9NP+1	$4.5000\times 10^{14}$
	PSO	5NP+2	$5.0000\times 10^{14}$
	DE	2NP	$1.0000\times 10^{14}$
	ABC	5NP−3	$2.4999\times 10^{14}$
	PSO-LMS	5NP+2N+2	$3.0200\times 10^{14}$
	MABC	5NP+2N−2	$3.0200\times 10^{14}$
	Proposed Algorithm	7NP+8	$2.2271\times 10^{14}$
Multiplication	GWO	15NP+1	$7.5000\times 10^{14}$
	PSO	5NP+2	$5.0000\times 10^{14}$
	DE	NP	$5.0000\times 10^{13}$
	ABC	4NP+3	$2.0001\times 10^{14}$
	PSO-LMS	5NP+4N	$3.0101\times 10^{14}$
	MABC	4NP+2N+4	$2.4201\times 10^{14}$
	Proposed Algorithm	7NP+7	$2.2271\times 10^{14}$

## Data Availability

No new data were created or analyzed in this study. Data sharing is not applicable to this article.

## References

[1] Mahbub U., Acharjee P.P., Fattah S.A. An acoustic echo cancellation scheme based on particle swarm optimization algorithm. Proceedings of the TENCON 2010—2010 IEEE Region 10 Conference.

[2] Mahbub U., Acharjee P.P., Fattah S.A. A time domain approach of acoustic echo cancellation based on particle swarm optimization. Proceedings of the IEEE International Conference on Electrical & Computer Engineering (ICECE 2010).

[3] Pichardo E., Anides E., Vazquez A., Garcia L., Avalos J.G., Sánchez G., Pérez H.M., Sánchez J.C. (2023). A Compact and High-Performance Acoustic Echo Canceller Neural Processor Using Grey Wolf Optimizer along with Least Mean Square Algorithms. Mathematics.

[4] Kimoto M., Asami T. (2016). Multichannel Acoustic Echo Canceler Based on Particle Swarm Optimization. Electron. Commun. Jpn..

[5] Tang J., Zhao X. Particle swarm optimization with adaptive mutation. Proceedings of the 2009 IEEE WASE International Conference on Information Engineering.

[6] Ratanavilisagul C., Kruatrachue B. Selective crossover base on fitness in multiswarm optimization. Proceedings of the International Conference on Emerging Trends in Computer and Image Processing (CIP’11).

[7] Chi Y., Cai G. Particle swarm optimization with opposition-based disturbance. Proceedings of the 2010 2nd International Asia Conference on Informatics in Control, Automation and Robotics (CAR 2010).

[8] Mauryan R., Thanushkodi K., Sakthisuganya A. (2012). Reactive power optimization using quantum particle swarm optimization. J. Comput. Sci..

[9] Lu S., Yu S. An improved particle swarm optimizer with attraction and repulsion. Proceedings of the 2012 7th International Conference on Computing and Convergence Technology (ICCCT).

[10] Arani B.O., Mirzabeygi P., Panahi M.S. (2013). An improved PSO algorithm with a territorial diversity-preserving scheme and enhanced exploration–exploitation balance. Swarm Evol. Comput..

[11] Zermani A., Manita G., Feki E., Mami A. (2023). Hardware implementation of particle swarm optimization with chaotic fractional-order. Neural Comput. Appl..

[12] Da Costa A.L., Silva C.A., Torquato M.F., Fernandes M.A. (2019). Parallel implementation of particle swarm optimization on FPGA. IEEE Trans. Circuits Syst. II Express Briefs.

[13] Shaikh U.T., Kalwar I.H., Memon T.D., Shaikh F. (2017). Design of IIR filter using PSO algorithm and its implementation in FPGA. Indian J. Sci. Technol..

[14] Tang Y., Wang Z., Fang J.A. (2011). Parameters identification of unknown delayed genetic regulatory networks by a switching particle swarm optimization algorithm. Expert Syst. Appl..

[15] Zhan Z.H., Zhang J., Li Y., Chung H.S.H. (2009). Adaptive particle swarm optimization. IEEE Trans. Syst. Man Cybern. Part B Cybern..

[16] lnternational Telecommunication Union ITU-T (2002). Digital Network Echo Cancellers. Standardization Sector of ITU.

[17] Rout N.K., Das D.P., Panda G. (2011). Particle swarm optimization based active noise control algorithm without secondary path identification. IEEE Trans. Instrum. Meas..

[18] Mirjalili S., Mirjalili S.M., Lewis A. (2014). Grey wolf optimizer. Adv. Eng. Softw..

[19] Krusienski D., Jenkins W. Adaptive filtering via particle swarm optimization. Proceedings of the The Thrity-Seventh Asilomar Conference on Signals, Systems & Computers.

[20] Reddy K.S., Sahoo S.K. (2015). An approach for FIR filter coefficient optimization using differential evolution algorithm. AEU-Int. J. Electron. Commun..

[21] Bansal J.C., Sharma H., Jadon S.S. (2013). Artificial bee colony algorithm: A survey. Int. J. Adv. Intell. Paradig..

[22] Krusienski D., Jenkins W. A particle swarm optimization-least mean squares algorithm for adaptive filtering. Proceedings of the Conference Record of the Thirty-Eighth Asilomar Conference on Signals, Systems and Computers.

[23] Ren X., Zhang H. (2022). An Improved Artificial Bee Colony Algorithm for Model-Free Active Noise Control: Algorithm and Implementation. IEEE Trans. Instrum. Meas..

[24] Maya X., Garcia L., Vazquez A., Pichardo E., Sanchez J.C., Perez H., Avalos J.G., Sanchez G. (2023). A high-precision distributed neural processor for efficient computation of a new distributed FxSMAP-L algorithm applied to real-time active noise control systems. Neurocomputing.

[25] Hasnat A., Bhattacharyya T., Dey A., Halder S., Bhattacharjee D. A fast FPGA based architecture for computation of square root and Inverse Square Root. Proceedings of the 2017 Devices for Integrated Circuit (DevIC).

[26] Paleologu C., Ciochina S., Benesty J. (2008). Variable step-size NLMS algorithm for under-modeling acoustic echo cancellation. IEEE Signal Process. Lett..

